# Effect of Intraoperative Nefopam on Postoperative Analgesia in Living Liver Donors Undergoing Laparoscopic Hepatectomy with Transversus Abdominis Plane Block: A Propensity Score-Matched Study

**DOI:** 10.3390/life15040590

**Published:** 2025-04-03

**Authors:** Min Suk Chae, Jin-Oh Jeong, Kyung Kwan Lee, Wonwoo Jeong, Young Wook Moon, Ji Young Min

**Affiliations:** 1 Department of Anesthesiology and Pain Medicine, Seoul St. Mary’s Hospital, College of Medicine, The Catholic University of Korea, Seoul 03312, Republic of Korea; shscms@catholic.ac.kr; 2 Wake Forest Institute for Regenerative Medicine, Wake Forest School of Medicine, Winston-Salem, NC 27157, USA; jijeong@wakehealth.edu (J.-O.J.); kylee@wakehealth.edu (K.K.L.);; 3 CGBIO USA, US Research and Production Team, Winston-Salem, NC 27101, USA; moon@cgbio.co.kr; 4 Department of Anesthesiology and Pain Medicine, Eunpyeong St. Mary’s Hospital, College of Medicine, The Catholic University of Korea, Seoul 03312, Republic of Korea

**Keywords:** hepatectomy, laparoscopic, living donors, liver transplantation, nefopam, nerve block, pain

## Abstract

Laparoscopic surgery reduces tissue trauma and accelerates recovery, but postoperative pain remains a concern. Opioids are effective but have adverse effects, highlighting the need for multimodal analgesia. Nefopam, a non-opioid analgesic, provides pain relief without respiratory depression or dependence. This study aims to investigate the efficacy of intravenous nefopam combined with a transversus abdominis plane (TAP) block in living liver donors undergoing laparoscopic hepatectomy. This retrospective cohort analysis was conducted on 452 adult living donors who underwent laparoscopic hepatectomy with a TAP block between August 2013 and August 2018 at a single tertiary medical center. After propensity score matching, 296 patients were included, with 148 in the nefopam group and 148 in the non-nefopam group. The primary outcomes assessed were pain scores using the Numeric Rating Scale (NRS) at 1, 4, 8, 12, and 24 h postoperatively, opioid consumption, postoperative nausea and vomiting, and nefopam-related adverse effects. Nefopam significantly reduced NRS at 1, 4, and 8 h postoperatively (*p* < 0.001) and decreased fentanyl use in the post-anesthesia care unit (26.0 ± 32.2 μg vs. 60.5 ± 37.9 μg, *p* < 0.001) and total intravenous patient-controlled analgesia volume (*p* < 0.001). The incidence of postoperative nausea and vomiting and severe opioid-related complications did not differ between groups. Nefopam-related side effects were mild and self-limiting. Nefopam combined with a TAP block effectively reduces postoperative pain and opioid consumption in living liver donors, supporting its role in multimodal analgesia. Further research is needed to explore its broader applications.

## 1. Introduction

Laparoscopic surgery has transformed modern surgical practice by offering advantages over open surgery, including smaller incisions, reduced tissue trauma, and faster recovery [1]. While these benefits often contribute to reduced postoperative pain, effective pain management remains a challenge due to factors such as trocar insertion, peritoneal stretching, and CO_2_ insufflation-induced referred shoulder pain. Previous studies have demonstrated that patients undergoing laparoscopic cholecystectomy frequently experience sharp somatic pain at the surgical site, along with diffuse, dull visceral pain [2,3]. Additionally, postoperative shoulder pain has been reported in 55.9% of patients, a higher incidence than initially anticipated [4].

In living donor laparoscopic hepatectomy, postoperative pain management presents unique challenges beyond those encountered in other laparoscopic procedures. This procedure involves extensive hepatic parenchymal transection, prolonged pneumoperitoneum, and graft extraction through a Pfannenstiel incision, leading to a multifaceted pain profile that encompasses somatic, visceral, and neuropathic components. Although the Pfannenstiel incision provides cosmetic benefits, it is also associated with significant postoperative discomfort due to deep tissue dissection [5]. Furthermore, hepatic mobilization and diaphragmatic irritation can contribute to referred pain in the shoulder and upper abdomen, further complicating pain control [6]. Given the physiological demands placed on donors and the need for an uncomplicated recovery, optimizing analgesia in living donor laparoscopic hepatectomy remains a critical priority in perioperative care [7].

Opioids are a mainstay of postoperative analgesia but are associated with well-documented adverse effects, including respiratory depression, nausea, vomiting, constipation, and the risk of dependence [8,9]. These concerns are particularly relevant in living donor laparoscopic hepatectomy, as donors typically lack prior opioid exposure, making them more susceptible to opioid-related side effects [10]. Additionally, hepatectomy alters hepatic metabolism, potentially leading to unpredictable opioid pharmacokinetics and an increased risk of drug accumulation and toxicity. These challenges have driven the adoption of multimodal analgesia strategies that integrate various analgesic techniques to optimize pain relief while minimizing opioid use [11]. Despite the routine administration of intravenous opioid-based regimens in conjunction with regional anesthesia such as the TAP block, many patients undergoing living donor laparoscopic hepatectomy still require substantial opioid doses postoperatively, suggesting that current strategies may be insufficient [12,13]. Furthermore, conventional non-opioid analgesics such as acetaminophen and nonsteroidal anti-inflammatory drugs (NSAIDs) may be unsuitable in this population due to their potential impact on hepatic function. This underscores the need for alternative non-opioid agents that are both safe and effective for pain management in living liver donors [14].

Nefopam, a centrally acting non-opioid analgesic, has emerged as a potential adjunct in multimodal pain management. By inhibiting the reuptake of serotonin (5-HT), norepinephrine (NE), and dopamine (DA), nefopam enhances descending pain modulation, reduces central sensitization, and attenuates nociceptive transmission in the spinal dorsal horn [15]. Unlike opioids, nefopam does not cause respiratory depression or dependence, making it a compelling option for living donor laparoscopic hepatectomy patients requiring effective pain control while minimizing opioid exposure [16,17]. However, despite its potential advantages, nefopam’s role in perioperative pain management remains underexplored, and its clinical utilization has been inconsistent. Given the persistent postoperative pain observed in this surgical population despite intravenous patient-controlled analgesia (IV-PCA) and the TAP block, nefopam may offer a beneficial addition to multimodal analgesia, potentially addressing an unmet need in current pain management protocols for living liver donors.

We hypothesize that nefopam may enhance postoperative pain relief, reduce opioid consumption, and facilitate a smoother recovery when combined with the TAP block in living donors undergoing laparoscopic hepatectomy. By investigating its impact in this patient population, this study aims to evaluate the analgesic efficacy of intravenous nefopam infusion and shed light on its role within multimodal pain management protocols, as well as its potential applicability in optimizing perioperative care for living liver donors.

## 2. Methods

### 2.1. Ethical Considerations

This retrospective propensity score-matched cohort study received approval from the Institutional Review Board and Ethics Committee of Seoul St. Mary’s Hospital (approval number: KC18RESI0485) on 16 August 2018. This study was conducted in compliance with the ethical principles outlined in the Declaration of Helsinki. Due to its retrospective design, the requirement for informed consent was waived by the Ethics Committee. To maintain transparency and methodological rigor, this study adhered to the Strengthening the Reporting of Observational Studies in Epidemiology (STROBE) guidelines.

### 2.2. Participant Population

This retrospective, propensity score-matched cohort study was conducted at a single tertiary medical center, analyzing electronic medical records from August 2013 to August 2018. Eligible participants included living liver donors aged 19 years or older who underwent elective laparoscopic hepatectomy and received a TAP block as part of their postoperative pain management strategy.

Patients were excluded if they were under 19 years of age or declined TAP block administration. Additionally, individuals with contraindications to the TAP block, including coagulopathy (international normalized ratio [INR] > 2.0 or platelet count < 50 × 10^9^/L [18]), were deemed ineligible. Patients with a documented allergy or hypersensitivity to nefopam were also excluded, as well as those with a history of seizures or psychosis, given nefopam’s potential to lower the seizure threshold and exacerbate psychiatric symptoms when used alongside certain medications. Further exclusions included open hepatectomy, hemodynamic instability, postoperative psychosis, or the need for reoperation, as these factors could influence postoperative pain outcomes. Patients who experienced severe postoperative nausea and vomiting (PONV), resulting in the discontinuation of IV-PCA within 24 h postoperatively, were also omitted. To ensure data integrity and minimize confounding variables, individuals with incomplete postoperative records or missing key data on pain scores, opioid consumption, or PONV outcomes were excluded. Lastly, patients who received alternative analgesic modalities, such as epidural analgesia, were not included, as these interventions could confound the assessment of nefopam’s analgesic effects.

**Figure 1:**
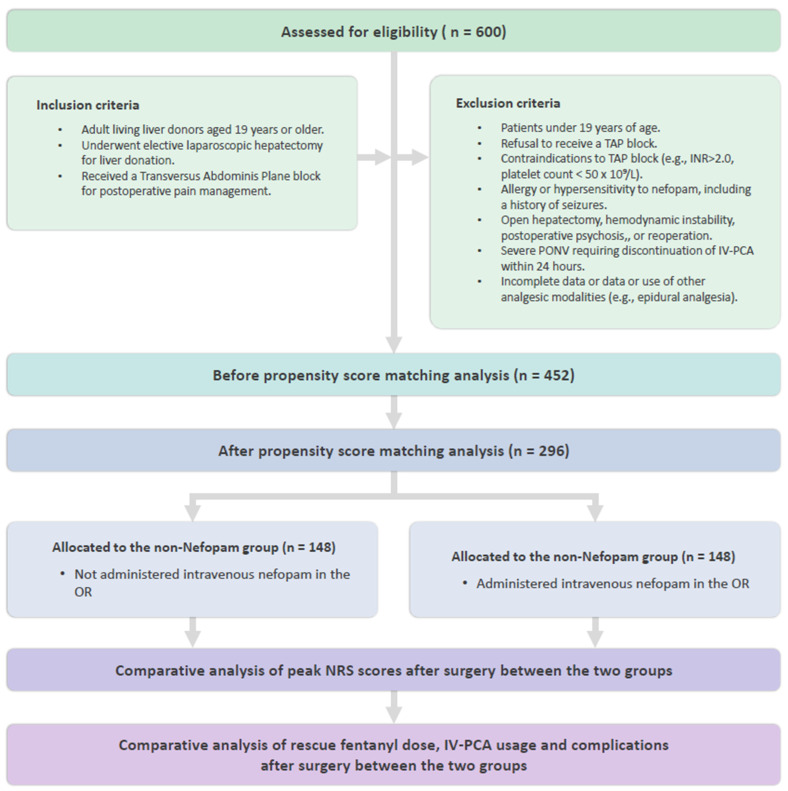
Consort diagram. TAP, transversus abdominis plane; INR, international normalized ratio; PONV, postoperative nausea and vomiting; IV-PCA, intravenous patient-controlled analgesia; NRS, Numeric Rating Scale; OR, operative room.

From an initial cohort of 600 patients, 452 met the eligibility criteria following the application of exclusion criteria. To achieve comparable baseline characteristics, propensity score matching was conducted, yielding a final cohort of 296 patients, evenly divided into the nefopam group (*n* = 148) and the non-nefopam group (*n* = 148) for the final analysis (Figure 1).

### 2.3. General Anesthesia and Intravenous Opioid Analgesia

General anesthesia was induced and maintained according to institutional protocols. Induction was performed with the intravenous propofol (1.5–2.5 mg/kg), while analgesia was achieved with a fentanyl bolus (1–2 μg/kg), followed by a continuous infusion of remifentanil (0.05–0.2 μg/kg/min). Rocuronium (0.6–1.2 mg/kg) was administered to facilitate neuromuscular blockade. Anesthesia was maintained with desflurane (4–6 vol%) and a continuous infusion of remifentanil at the same dosage range. Perioperative monitoring was standardized for all patients. Invasive arterial blood pressure (ABP), including mean arterial pressure (MAP), was continuously monitored via a radial artery catheter, while central venous pressure (CVP) was measured using a preoperatively placed central venous catheter. To minimize blood loss and reduce the need for transfusions, CVP was maintained at ≤5 mmHg throughout the procedure, with fluid administration carefully balanced. Upon completion of surgery, all donors were extubated in the operating room and transferred to the intensive care unit (ICU) for close monitoring, particularly for postoperative hemorrhage and hemodynamic stability. To prevent PONV, palonosetron hydrochloride (0.075 mg) was administered preemptively. For patients who developed PONV postoperatively, ondansetron (4 mg) was given as needed.

Postoperative analgesia was primarily managed with IV-PCA. The IV-PCA device (AutoMed 3200, Acemedical, Seoul, Republic of Korea) contained fentanyl (1000 μg) and ramosetron (0.3 mg), diluted to 100 mL with normal saline. The device was programmed for a continuous infusion of 1 mL/h, with a 1 mL bolus available at a 10 min lockout interval. Patients were instructed to self-administer a bolus dose if their Numeric Rating Scale (NRS) pain score reached 4 or higher (0 = no pain; 10 = worst imaginable pain), ensuring both baseline pain control and immediate relief of breakthrough pain.

For patients with insufficient pain relief (NRS ≥ 4) despite IV-PCA, an additional 50 μg fentanyl dose was administered by an attending physician uninvolved in the research. Both cumulative IV-PCA fentanyl consumption and total rescue fentanyl doses were recorded at each evaluation to quantify opioid use. Patients were also closely monitored for opioid-related adverse effects, including respiratory depression and PONV, with timely interventions as necessary.

### 2.4. Surgery

Laparoscopic hepatectomy for living liver donation utilizes minimally invasive techniques, beginning with small abdominal incisions for trocar placement to accommodate laparoscopic instruments. CO_2_ insufflation is performed to expand the abdominal cavity, enhancing surgical field visualization. The liver is mobilized by dissecting the falciform, coronary, and triangular ligaments. Intraoperative ultrasound imaging is employed to map the hepatic anatomy, identify vascular and biliary structures, and confirm resection margins. Hepatic parenchymal transection is carried out using advanced surgical tools, such as a harmonic scalpel or cavitron ultrasonic surgical aspirator (CUSA), ensuring precise dissection and preservation of critical structures. Major vascular and biliary structures are secured using ligatures or clips before completing the resection. The liver graft is extracted through a Pfannenstiel incision, a horizontal incision above the pubic symphysis, designed to preserve graft integrity and facilitate safe removal. Following graft retrieval, the surgical site is thoroughly inspected for bleeding and bile leaks before incision closure [19].

### 2.5. TAP Block

To manage postoperative pain associated with the Pfannenstiel incision, a bilateral TAP block was administered immediately after surgery by a single experienced anesthesiologist, ensuring consistency and objectivity. A linear ultrasound probe (Affiniti 70 Ultrasound System, Philips, Amsterdam, The Netherlands) was positioned along the mid-axillary line between the iliac crest and the lower costal margin, allowing clear visualization of the external oblique, internal oblique, and transversus abdominis muscle layers. A 21-gauge, 8.5 mm sterile block needle (Echoplex; Vygon Co., Paris, France) was inserted medially to laterally using an in-plane ultrasound-guided approach, ensuring continuous needle tip visualization. After reaching the fascial plane between the internal oblique and transversus abdominis muscles, hydrodissection with a test dose of saline confirmed correct needle placement. Once verification was complete, 20 mL of 0.375% ropivacaine (Mitsubishi Tanabe Pharm. Co., Osaka, Japan) was incrementally injected on each side under ultrasound guidance, ensuring uniform anesthetic spread and procedural accuracy [20].

### 2.6. Intravenous Nefopam Infusion

As part of the multimodal analgesia regimen, nefopam (20 mg, diluted in 100 mL of normal saline; Myungmoon Pharm. Co., Seoul, Republic of Korea) was administered intravenously over 15 min during skin closure. Patients who received nefopam infusion were closely monitored in the post-anesthesia care unit (PACU) for potential side effects, including nausea, vomiting, dizziness, excessive sweating, tachycardia, and hypertension. Any adverse reactions were promptly addressed to ensure patient safety [21].

### 2.7. Assessment of Primary and Secondary Outcomes

The primary outcomes of this study included postoperative pain intensity, opioid consumption, the incidence of PONV, and nefopam-related adverse effects. Pain intensity was assessed using the Numeric Rating Scale (NRS) at 1, 4, 8, 12, and 24 h postoperatively. At the study institution, this assessment schedule follows the standard operating protocol (SOP) for all surgical patients: NRS scores are routinely recorded at 1 and 4 h in the PACU, and at 8, 12, and 24 h in either the ICU or general ward, depending on the patient’s postoperative recovery location. This schedule is consistently applied to patients regardless of their inclusion in research protocols. Opioid consumption was quantified by recording fentanyl administration in the PACU and ICU (μg), along with the total volume of IV-PCA infused (mL).

As secondary outcomes, the incidence of PONV within the first 24 postoperative hours was documented. Additionally, nefopam-related adverse effects, including nausea, vomiting, dizziness, excessive perspiration, tachycardia, and hypertension, were systematically monitored and recorded.

### 2.8. Data Collection

The clinical variables in this study included both preoperative and intraoperative parameters. Preoperative variables encompassed demographic data (sex, age, and body mass index), comorbidities such as diabetes mellitus and hypertension, history of abdominal surgery, liver fat percentage, and baseline laboratory values including white blood cell count, hemoglobin, platelet count, urea nitrogen, creatinine, albumin, liver enzymes (Aspartate Aminotransferase [AST], Alanine Aminotransferase [ALT]), total bilirubin, electrolytes (sodium, potassium, chloride), and coagulation markers (INR, Prothrombin Time [PT], Activated Partial Thromboplastin Time [aPTT]). Vital signs such as MBP and heart rate (HR) were also recorded preoperatively. Intraoperative variables included operation duration, blood loss, transfusion requirements (Packed Red Blood Cells [pRBC] and Fresh Frozen Plasma [FFP]), hourly fluid infusion rates, and urine output. Average intraoperative vital signs, such as MBP and HR, were also monitored. These variables were compared between the nefopam and non-nefopam groups both before and after PS matching to ensure balanced baseline characteristics.

### 2.9. Statistical Analysis

The Shapiro–Wilk test was employed to evaluate the normality of continuous variables. Categorical variables were presented as numbers and percentages, while continuous variables were expressed as means with standard deviations (SDs) or medians with interquartile ranges (IQRs), depending on their distribution. PS matching was performed using a logistic regression model that included the following covariates: age, sex, body mass index (BMI), history of abdominal surgery, comorbidities (diabetes mellitus and hypertension), liver fat percentage, preoperative laboratory values (white blood cell count, hemoglobin, platelet count, urea nitrogen, creatinine, albumin, AST, ALT, total bilirubin, sodium, potassium, chloride, INR, PT, aPTT), preoperative vital signs (mean blood pressure and heart rate), and intraoperative variables (operation duration, blood loss, transfusion amounts, fluid infusion rate, urine output, average intraoperative mean blood pressure, and heart rate). These covariates were selected based on their potential influence on postoperative pain and opioid consumption. PS score matching was conducted using a 1:1 nearest-neighbor algorithm without replacement. For statistical comparisons, categorical data were analyzed using either the chi-squared test or Fisher’s exact test, while continuous data were evaluated with Student’s *t*-test or the Mann–Whitney U test, based on the normality of their distribution. A two-tailed *p*-value of less than 0.05 was considered statistically significant. Data analyses were performed using R software (version 2.10.1; R Foundation for Statistical Computing, Vienna, Austria), and graphical outputs were generated with Microsoft Excel (Microsoft Corporation, Redmond, WA, USA).

## 3. Results

### 3.1. Demographic Features of the Study Population

Table 1 presents the baseline preoperative and intraoperative characteristics of patients in the nefopam and non-nefopam groups, both before and after propensity score matching. Before matching, 301 patients were included in the non-nefopam group and 151 in the nefopam group. A significant difference was observed in AST levels, which were higher in the nefopam group than in the non-nefopam group (21.1 ± 8.6 U/L vs. 19.9 ± 6.3 U/L, *p* = 0.009). However, other baseline characteristics, including age, sex, BMI, comorbidities (diabetes mellitus and hypertension), history of abdominal surgery, and laboratory parameters such as hemoglobin, platelet count, liver fat percentage, and electrolytes, did not differ significantly between the groups.

After propensity score matching, 148 patients were included in each group, resulting in well-balanced baseline characteristics. No significant differences were found between the nefopam and non-nefopam groups in terms of demographic factors (age, sex, and BMI), clinical variables (comorbidities, history of abdominal surgery, and laboratory values such as hemoglobin, platelet count, AST, ALT, electrolytes, and creatinine), or perioperative variables (operation duration, blood loss, transfusion requirements, and intraoperative vital signs, including mean blood pressure and heart rate).

### 3.2. Postoperative Pain, Opioid Consumption, and Complications

**Figure 2:**
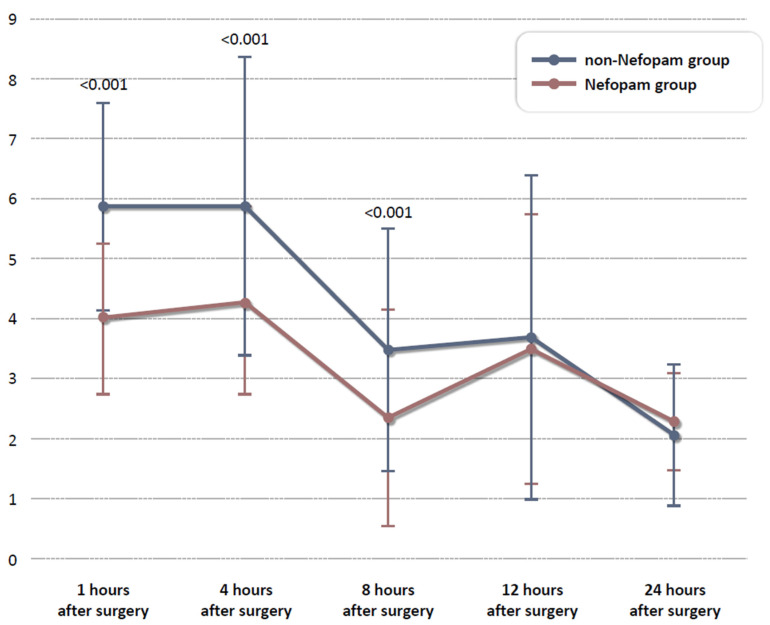
Comparison of NRS scores between the nefopam and non-nefopam groups over time. The vertical axis represents time, while the horizontal axis represents the Numeric Rating Scale (NRS). Data are presented as mean ± standard deviation (SD). The non-nefopam group is shown in blue, and the nefopam group is shown in red.

The postoperative outcomes of the nefopam and non-nefopam groups, each comprising 148 propensity score-matched patients, are summarized in Table 2. Pain intensity, assessed using the NRS, was significantly lower in the nefopam group compared to the non-nefopam group at 1 h (4.0 ± 1.2 vs. 5.9 ± 1.7, *p* < 0.001), 4 h (4.3 ± 1.5 vs. 5.9 ± 2.5, *p* < 0.001), and 8 h (2.3 ± 1.8 vs. 3.5 ± 2.0, *p* < 0.001) postoperatively, as illustrated in Figure 2. However, no significant differences in pain scores were observed at 12 h (3.5 ± 2.3 vs. 3.7 ± 2.7, *p* = 0.514) or 24 h (2.3 ± 0.8 vs. 2.1 ± 1.2, *p* = 0.082).

Opioid consumption was significantly lower in the nefopam group. Fentanyl usage in the PACU was significantly lower (26.0 ± 32.2 μg vs. 60.5 ± 37.9 μg, *p* < 0.001), as was the total IV-PCA volume (44.0 ± 19.2 mL vs. 71.8 ± 28.9 mL, *p* < 0.001). Although fentanyl consumption in the ward was lower in the nefopam group (25.3 ± 25.1 μg vs. 29.4 ± 24.7 μg), the difference was not statistically significant (*p* = 0.162). The incidence of postoperative nausea and vomiting was lower in the nefopam group (12.8% vs. 19.6%), though the difference was not statistically significant (*p* = 0.115).

Beyond PONV, no severe complications were observed. Notably, there were no cases of major opioid-related complications, including respiratory depression, excessive sedation, or ileus, in either group. Additionally, no other potential opioid-related side effects, such as pruritus or urinary retention, were reported.

### 3.3. Nefopam-Related Side Effects

In this study, patients who received nefopam were closely monitored for potential adverse effects, excluding nausea and vomiting, which are commonly associated with postoperative conditions. Among the nefopam group, mild side effects were observed in 7 out of 148 patients (4.7%), including dizziness in 4 patients (2.7%), tachycardia in 2 patients (1.4%), and excessive sweating in 1 patient (0.7%). These symptoms were mild, transient, and resolved spontaneously without requiring additional medical intervention. Additionally, all living liver donors spent one day in the ICU before being uneventfully transferred to the general ward.

## 4. Discussion

This study demonstrates that incorporating nefopam alongside a TAP block significantly improves early postoperative analgesia and reduces opioid consumption in living donors undergoing laparoscopic liver donation surgery. The nefopam group required significantly lower doses of fentanyl in the PACU and showed a marked reduction in IV-PCA usage compared to the non-nefopam group, reinforcing its opioid-sparing potential. While the incidence of PONV was lower in the nefopam group, the difference was not statistically significant. Importantly, nefopam-related side effects, including mild dizziness, tachycardia, and excessive sweating, were infrequent, transient, and resolved without medical intervention, confirming its safety and tolerability in this population. These findings suggest that nefopam, when combined with regional anesthesia, provides enhanced pain relief while minimizing opioid exposure, ultimately contributing to safer and faster recovery for living liver donors.

Building on these clinical observations, the pharmacological profile of nefopam may explain its observed efficacy in this context. Nefopam’s analgesic effects in the immediate postoperative period, when nociceptive input is most intense, may be attributed to its multimodal mechanism—namely, monoamine reuptake inhibition, NMDA receptor antagonism, and modulation of sodium and calcium channels [22,23]. Given its rapid onset (within 30 min) and peak effect around 60 min post-administration [24], the timing of nefopam use in this study appears appropriate for effective early postoperative pain control. This is particularly relevant for living liver donors, who face distinct postoperative pain challenges due to the extensive nature of hepatectomy, psychological stress, and the absence of prior chronic pain exposure [10]. By addressing both the intensity and complexity of postoperative pain in this population, nefopam could serve as an integral component of multimodal analgesia protocols. This study’s findings further support its integration into perioperative pain management strategies to optimize analgesic efficacy while minimizing opioid reliance in liver surgery patients.

This mechanism-based rationale is further supported by previous studies demonstrating nefopam’s opioid-sparing effects in various surgical settings. A meta-analysis reported that intravenous nefopam administration significantly reduces postoperative pain scores and opioid consumption. Similarly, a randomized controlled trial found that nefopam, when included in PCA regimens, provided comparable pain relief to opioid-only protocols while significantly lowering opioid-related adverse effects [25]. While laparoscopic liver resection offers advantages such as reduced surgical trauma, faster recovery, and lower complication rates, postoperative pain remains a challenge due to prolonged pneumoperitoneum, extensive hepatic mobilization, and the need for subcostal or midline incisions for specimen retrieval [26]. Nefopam has demonstrated effectiveness across a range of surgical procedures, from minimally invasive robot-assisted laparoscopic cholecystectomy to more extensive hepatic resections. In hepatic surgery, a comparative study with propacetamol confirmed nefopam’s analgesic efficacy while maintaining hemodynamic stability, reinforcing its role in multimodal analgesia. Furthermore, a randomized controlled trial on single-port robotic cholecystectomy demonstrated that intraoperative nefopam administration significantly reduced right upper quadrant pain (median 5 vs. 9, *p* < 0.001), lowered postoperative opioid consumption (PACU fentanyl: median 0 vs. 50 μg, *p* = 0.002), and improved postoperative nausea and vomiting (*p* = 0.006), contributing to enhanced recovery [27,28].

This study further investigates the role of nefopam as an adjunct to a TAP block in multimodal analgesia in living donors undergoing laparoscopic hepatectomy. While the TAP block effectively manages somatic pain in laparoscopic liver surgery, its limited efficacy in controlling visceral and diaphragmatic pain necessitates the use of systemic analgesics [26,29]. The results of this study suggest that nefopam can enhance early postoperative pain relief beyond what a TAP block alone provides. Furthermore, the significant reduction in fentanyl consumption in the nefopam group suggests that it may contribute to minimizing opioid reliance and associated adverse effects. Considering that living liver donors face challenges associated with opioid metabolism in hepatic resection, including unpredictable clearance and an increased risk of drug accumulation and toxicity, the opioid-sparing effect of nefopam supports its role as a safer alternative for patients with altered hepatic function [30]. Moreover, nefopam undergoes minimal hepatic metabolism [31], reinforcing its suitability as a non-opioid analgesic for patients with altered liver function. Despite these advantages, the benefits of nefopam appear to be time-limited. No significant differences in pain scores were observed between groups at 12 and 24 h postoperatively. This may be explained by nefopam’s relatively short elimination half-life of approximately 3 to 5 h [32], which limits its duration of action following a single dose. This suggests that sustained pain relief may require additional strategies such as repeated dosing, continuous infusion, or the incorporation of complementary analgesic modalities within a comprehensive multimodal pain management framework.

Based on these findings, changes in clinical practice were implemented during the study period (2013–2018). At the time, the standard postoperative pain management protocol involved IV-PCA with fentanyl and a bilateral single-shot TAP block. Epidural-PCA was not routinely used due to concerns related to coagulopathy and hemodynamic instability in donor hepatectomy patients. Following this study, a revised multimodal analgesic protocol was adopted, with nefopam now frequently incorporated as a non-opioid adjuvant to reduce opioid requirements while maintaining effective pain control. Additionally, a follow-up study is underway to evaluate long-term outcomes in this patient population, aiming to determine whether the early analgesic benefits of nefopam contribute to improved recovery trajectories and overall quality of life.

While the analgesic benefits were evident, the overall incidence of PONV was not significantly different. PONV is a multifactorial condition influenced by numerous variables beyond opioid exposure, including patient-specific characteristics (e.g., female sex, non-smoking status, and a history of PONV) as well as perioperative factors such as anesthetic type and dose (particularly inhalational agents), surgical duration, postoperative fluid management, and the use of prophylactic antiemetics [33,34]. This lack of significant difference may also reflect the influence of these dominant risk factors or limitations in this study’s assessment method, which focused solely on overall incidence rather than symptom severity or the need for rescue antiemetics [35,36]. Moreover, nefopam itself has been associated with nausea and vomiting in some individuals, particularly at higher doses. Its mechanism—particularly its serotonergic and dopaminergic activity—has been implicated in these side effects [37,38]. These findings highlight the complex interplay of multiple risk factors contributing to PONV and underscore the need for further investigations to better define nefopam’s role in postoperative nausea prevention. Future studies should explore optimal antiemetic strategies when incorporating nefopam into multimodal analgesia protocols.

This study has several limitations that should be carefully considered when interpreting its findings. First, the retrospective design may introduce selection bias, as reliance on historical records can lead to systematic differences between comparison groups that are not fully accounted for—even with propensity score matching—since unmeasured confounding variables may persist, as documented in epidemiological methodology studies. Second, this study was conducted at a single center with a relatively homogeneous patient population, limiting the external validity of the findings. Institutional practices, regional demographics, and clinical protocols may differ across settings, potentially affecting the generalizability of the results [39]. Third, while this study focused on evaluating the opioid-sparing effects and immediate safety profile of nefopam, the limited long-term data introduce uncertainties regarding its sustained benefits and potential delayed adverse events [40]—an issue raised in comparable pharmacological research [41]. Finally, key postoperative outcomes such as patient satisfaction and functional recovery were not assessed [42,43]. These dimensions are critical for a comprehensive evaluation of pain management interventions. A further consideration is that reliance on self-reported, transient side effects may have led to underreporting of mild symptoms due to inherent recall and reporting biases. Future prospective, multicenter studies with larger sample sizes and comprehensive outcome measures are needed to validate these findings and further elucidate nefopam’s role in multimodal pain management strategies for living donors.

## 5. Conclusions

In conclusion, this study demonstrates that nefopam, when used with a TAP block, enhances postoperative pain management in living liver donors undergoing laparoscopic hepatectomy. Nefopam was associated with improved pain control and a significant reduction in opioid consumption in this study, suggesting its potential role as a complementary analgesic in multimodal pain management. These findings suggest that integrating nefopam with regional anesthesia techniques may improve recovery by reducing opioid-related risks. Further research is needed to evaluate its long-term benefits and broader applicability across surgical populations.

## Figures and Tables

**Table 1:** Pre- and intraoperative variables in the nefopam and non-nepofam groups before and after propensity score matching.

	Before Propensity Score Matching				After Propensity Score Matching			
	Non-Nepofam (*n* = 301)	Nefopam (*n* = 151)	*p* Value	SD	Non-Nepofam (*n* = 148)	Nefopam (*n* = 148)	*p* Value	SD
**Preoperative variables**								
Sex; *n* (%)	176 (58.5%)	99 (65.6%)	0.145	−0.149	103 (69.6%)	97 (65.5%)	0.456	0.085
Age; years	34.5 ± 11.3	35.6 ± 12.4	0.345	0.089	35.7 ± 12.0	35.3 ± 12.2	0.803	−0.028
Body mass index; kg/m^2^	23.5 ± 3.0	23.8 ± 3.1	0.385	0.086	23.7 ± 3.1	23.7 ± 3.1	0.902	0.014
Liver fatty percentage; %	4.4 ± 5.9	4.7 ± 7.9	0.618	0.042	3.9 ± 4.5	4.2 ± 5.4	0.633	0.035
Diabetes mellitus; *n* (%)	4 (1.3%)	2 (1.3%)	>0.999	0.000	2 (1.4%)	2 (1.4%)	>0.999	0.000
Hypertension; *n* (%)	11 (3.7%)	8 (5.3%)	0.411	0.073	9 (6.1%)	8 (5.4%)	0.803	−0.030
History of abdominal surgery; *n* (%)	49 (16.3%)	30 (19.9%)	0.343	0.090	26 (17.6%)	29 (19.6%)	0.654	0.051
Mean blood pressure; mmHg	90.8 ± 10.6	91.5 ± 10.5	0.512	0.066	92.4 ± 10.0	91.7 ± 10.2	0.562	−0.065
Heart rate; beats/min	76.3 ± 10.7	76.9 ± 11.7	0.605	0.049	75.8 ± 9.5	76.5 ± 11.2	0.521	0.067
Laboratory values								
WBC count; ×10^9^/L	6.4 ± 1.6	6.6 ± 2.0	0.192	0.112	6.5 ± 1.7	6.5 ± 1.8	0.981	0.002
Hemoglobin; g/dL	14.1 ± 1.6	14.3 ± 1.5	0.244	0.125	14.5 ± 1.6	14.3 ± 1.5	0.421	−0.096
Platelet count; ×10^9^/L	243.6 ± 53.9	236.5 ± 53.8	0.187	−0.132	234.6 ± 48.5	237.6 ± 53.5	0.607	0.057
Urea nitrogen; mg/dL	13.6 ± 3.5	13.6 ± 3.6	0.840	−0.020	13.6 ± 3.6	13.5 ± 3.4	0.790	−0.030
Creatinine; mg/dL	0.8 ± 0.2	0.8 ± 0.2	0.111	0.139	0.8 ± 0.1	0.8 ± 0.2	0.582	−0.049
Albumin; g/dL	4.4 ± 0.4	4.4 ± 0.4	0.643	0.045	4.4 ± 0.3	4.4 ± 0.3	0.734	0.036
AST; U/L	19.9 ± 6.3	21.1 ± 8.6	0.009 *	0.242	21.0 ± 7.0	21.3 ± 6.8	0.719	0.034
ALT; U/L	19.4 ± 10.1	21.1 ± 9.9	0.094	0.169	20.3 ± 10.4	21.0 ± 9.9	0.557	0.070
Total bilirubin; mg/dL	0.6 ± 0.3	0.6 ± 0.3	0.142	−0.151	0.6 ± 0.3	0.6 ± 0.3	0.639	−0.053
Sodium; mmol/L	140.9 ± 1.8	141.2 ± 2.1	0.221	0.110	141.1 ± 1.9	141.2 ± 2.1	0.640	0.052
Potassium; mmol/L	4.2 ± 0.3	4.2 ± 0.3	0.149	−0.143	4.2 ± 0.3	4.2 ± 0.3	0.857	0.020
Chloride; mmol/L	104.6 ± 2.1	104.6 ± 2.4	0.933	−0.008	104.5 ± 2.0	104.6 ± 2.4	0.696	0.042
INR	1.02 ± 0.07	1.02 ± 0.09	0.778	0.025	1.02 ± 0.06	1.02 ± 0.07	0.677	−0.036
PT; sec	11.4 ± 0.8	11.3 ± 1.0	0.878	−0.013	11.3 ± 0.8	11.3 ± 0.8	0.741	−0.030
aPTT; sec	27.9 ± 4.1	28.0 ± 5.0	0.929	0.008	27.7 ± 3.9	27.8 ± 4.2	0.99	0.001
**Intraoperative variables**								
Operation duration; min	253.5 ± 58.8	261.6 ± 54.0	0.157	0.150	261.0 ± 62.1	260.8 ± 54.1	0.972	−0.004
PRBC transfusion; units	0.3 ± 0.7	0.4 ± 1.0	0.157	0.130	0.3 ± 0.8	0.4 ± 1.0	0.651	0.048
FFP transfusion; units	1.3 ± 1.1	1.3 ± 1.0	0.956	0.006	1.4 ± 1.1	1.3 ± 1.0	0.506	−0.079
Hourly fluid infusion; mL/kg/hr	8.7 ± 3.4	8.6 ± 3.3	0.764	−0.031	8.3 ± 3.2	8.6 ± 3.3	0.418	0.094
Hourly urine output; mL/kg/h	1.4 ± 0.9	1.4 ± 1.0	0.984	0.002	1.3 ± 1.0	1.4 ± 1.0	0.647	0.055
Blood loss; mL	587.5 ± 367.2	637.8 ± 493.9	0.224	0.102	646.3 ± 410.0	637.2 ± 497.8	0.863	−0.018
**Average vital signs**								
Mean blood pressure; mmHg	84.2 ± 9.8	84.4 ± 9.7	0.766	0.030	85.4 ± 9.6	84.5 ± 9.7	0.430	−0.092
Heart rate; beats/min	76.0 ± 10.3	75.7 ± 9.3	0.707	−0.040	74.9 ± 9.5	75.4 ± 8.7	0.640	0.054

Values are expressed as mean ± standard deviation and number (percentage). SD, standard deviation; WBC, white blood cell; AST, aspartate aminotransferase; ALT, alanine aminotransferase; INR, international normalized ratio; PT, prothrombin time; aPTT, activated partial thromboplastin time; PRBC, packed red blood cell; FFP fresh frozen plasma. * *p* < 0.05.

**Table 2:** Postoperative pain scale, opioid dose, and complications in nefopam and non-nefopam group after propensity score matching.

Parameters	Non-Nefopam (*n* = 148)	Nefopam (*n* = 148)	*p* Value
Numeric rating scale (NRS)			
1 h after surgery	5.9 ± 1.7	4.0 ± 1.2	<0.001 **
4 h after surgery	5.9 ± 2.5	4.3 ± 1.5	<0.001 **
8 h after surgery	3.5 ± 2.0	2.3 ± 1.8	<0.001 **
12 h after surgery	3.7 ± 2.7	3.5 ± 2.3	0.514
24 h after surgery	2.1 ± 1.2	2.3 ± 0.8	0.082
Fentanyl dose; μg			
In the PACU	60.5 ± 37.9	26.0 ± 32.2	<0.001 **
In the ward	29.4 ± 24.7	25.3 ± 25.1	0.162
IV-PCA; mL	71.8 ± 28.9	44.0 ± 19.2	<0.001 **
PONV; %	29 (19.6%)	19 (12.8%)	0.115

Values are expressed as mean (±standard deviation) and number (percentage). ** *p* < 0.001. PACU, post-anesthesia care unit; IV-PCA, intravenous patient-controlled analgesia; PONV, postoperative nausea and vomiting.

## Data Availability

Data are contained within the article.
